# Persistent bilateral anterior hip pain in a young adult due to meralgia paresthetica: a case report

**DOI:** 10.1186/1757-1626-1-396

**Published:** 2008-12-15

**Authors:** Vijay D Shetty, Gautam M Shetty

**Affiliations:** 1 Department of Orthopaedic Surgery, L.H Hiranandani Hospital, Powai, Mumbai 400076, India

## Abstract

**Background:**

We describe a case report where a young woman presented with persistent bilateral anterior hip pain whose diagnosis was obscure for many months.

**Case presentation:**

The symptoms started three months after she underwent laparoscopic surgery, with entry portals on both iliac regions of her abdomen. After a thorough clinical examination, a working diagnosis of "Meralgia paresthetica" was made. She responded well to diagnostic block supplemented with local steroids.

**Conclusion:**

To our knowledge, this is the first ever case report of a bilateral meralgia paresthetica presenting as bilateral persistent anterior hip pain following a laparoscopic procedure.

## Background

The exact incidence of meralgia parasthetica is unknown owing to the difficulty in making a confirmed diagnosis. The possibility that this is the source of anterior hip pain should be considered when other sources have been discounted.[1] We present a case of persistent bilateral anterior hip pain, due to meralgia paresthetica, which responded well with local injection of anaesthetic and steroid. To our knowledge, this is the first case report of a bilateral meralgia paresthetica presenting as bilateral persistent anterior hip pain.

## Case presentation

A 26-year-old woman, homemaker, was referred to our department with persistent bilateral anterior hip pain of insidious onset of 9 months duration. Approximately 3 months before the onset of her symptoms, she underwent bilateral laparoscopic surgery for ovarian cysts. She saw several physicians and surgeons and underwent various modalities of investigations such as x-rays, ultrasound and MRI scan of her hips (all of which were reported normal) before she was referred to our clinic. Clinically she presented to us with vague pain in front of her both hips, worse on hip flexion (sitting), with occasional radiation to the level of her knees on both sides, associated with burning sensation and a limp on walking. The findings on clinical examination of both hips included mild anterior joint line tenderness, positive impingement sign with satisfactory joint range of motion. A pelvic examination and a repeat abdomen and pelvic ultrasound was performed which ruled out any active pathology of the pelvic organs. Manipulation under anaesthesia and injection, to rule out intra-articular hip pathology, gave no symptomatic relief.

Once most common causes of anterior hip pain and extra-skeletal causes were ruled out in this patient, we suspected the possibility of meralgia paresthetica, in view of her symptoms and signs. Nerve conduction study was normal. Since pain became a major issue for this patient, we decided to do a diagnostic block of the lateral cutaneous nerve of the thigh. The area 1 cm below and medial to the anterior superior iliac spine along the inguinal ligament was infiltrated with 3 cc of bupivacaine and 40 mg of triamcinolone on either side. The patient responded very well to this procedure and pain relief was instant. At the time of writing this article, twelve months after the procedure, patient remained symptom free.

## Discussion

**Figure 1:**
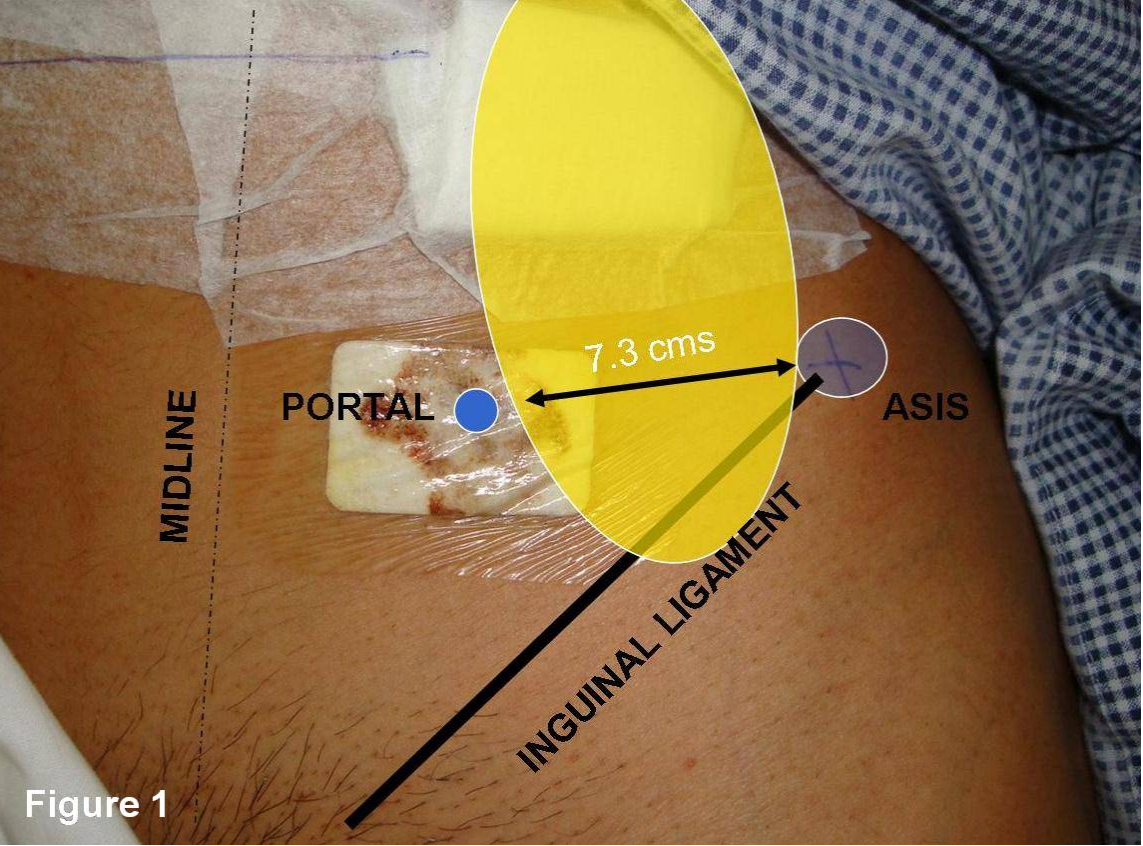
The typical site of portal for laparoscopic procedure shows its proximity to the course of the lateral femoral cutaneous nerve (large shaded oval area) that may travel up to 7.3 cm medial to the anterior superior iliac spine (ASIS).

Entrapment of the lateral femoral cutaneous nerve of thigh as a source of anterolateral thigh pain, has been recognised for more than a century. Despite this historic recognition, its diagnosis and treatment, today, is often delayed as the diagnosis is rarely considered.[2] Although the etiology of this condition can be varied, it has been reported after a number of surgical procedures such as hernia repair, open abdominal procedures and orthopaedic procedures, such as anterior iliac-crest bone-graft harvesting and anterior pelvic procedures. [3] Meralgia parasthetica, after laparoscopic procedures, may occur due to anatomic variations in the course of the nerve, extensive retroperitoneal dissection or patient position during surgery. [4-6] Studies have shown that the lateral cutaneous nerve may travel up to approximately 7.3 cm medial to the anterior superior iliac spine,[7,8] and most laparoscopic portals are made around this area [Figure 1].

If the physician is not familiar with this disorder and the involved anatomy, the search for a diagnosis can result in unnecessarily expensive tests and consultations. The diagnosis of meralgia parasthetica is quite straightforward once it has been considered.[3] Relief of pain and paresthesia after injection of a local anaesthetic agent is helpful in establishing the diagnosis.[3] Treatment of meralgia paresthetica has been somewhat controversial.[9] Literature suggests that most cases respond well for a conservative treatment of local injection.[3] If intractable pain persists, despite such measures, surgical intervention such as neurolysis, transposition and neurectomy can be considered.[3,9]

## Consent

Written informed consent was obtained from the patient for publication of this case report and accompanying images. A copy of the written consent is available for review by the Editor-in-Chief of this journal.

## Competing interests

The authors declare that they have no competing interests.

## Authors' contributions

GS analyzed and interpreted the patient data and VDS was a major contributor in writing the manuscript. All authors read and approved the final manuscript.
